# RETRACTION: Long Noncoding RNA AFAP1-AS1 Promotes Cell Proliferation and Metastasis via the miR-155-5p/FGF7 Axis and Predicts Poor Prognosis in Gastric Cancer

**DOI:** 10.1155/dim/9878012

**Published:** 2025-02-22

**Authors:** Disease Markers

RETRACTION: H-W. Ma, D-Y. Xi, J-Z. Ma, M. Guo, L. Ma, D-H. Ma, P-W. Li, and C-A. Guo. “Long Noncoding RNA AFAP1-AS1 Promotes Cell Proliferation and Metastasis via the miR-155-5p/FGF7 Axis and Predicts Poor Prognosis in Gastric Cancer” *Disease Markers*, no. 2020 (2020): https://doi.org/10.1155/2020/8140989

The above article, published online on 21 January 2020 in Wiley Online Library (wileyonlinelibrary.com), has been retracted by agreement between the handling editor, Robert Pichler; and John Wiley & Sons Ltd.

The retraction has been agreed following communication received from the authors indicating that they consider the contents to be unreliable, in part due to an error in Figure 3A,B. Additional concerns were also raised on PubPeer [1] regarding similarities detected between the si-black results for different cell types in Figure 2E. As such, we no longer have confidence in the integrity of the results presented.

The authors did not respond to our notice regarding the retraction.
